# Scoping Review of Japanese Encephalitis Virus Transmission Models

**DOI:** 10.1155/tbed/9880670

**Published:** 2025-01-27

**Authors:** Troy A. Laidlow, Erin S. Johnston, Ruth N. Zadoks, Michael Walsh, Mafalda Viana, Kerrie E. Wiley, Balbir B. Singh, Francesco Baldini, Himani Dhanze, Cameron Webb, Victoria J. Brookes

**Affiliations:** ^1^Sydney School of Veterinary Science, Faculty of Science, The University of Sydney, Camperdown, New South Wales, Australia; ^2^Sydney Infectious Diseases Institute, Faculty of Medicine and Health, The University of Sydney, Camperdown, New South Wales, Australia; ^3^School of Biodiversity, One Health and Veterinary Medicine, University of Glasgow, Glasgow, UK; ^4^Sydney School of Public Health, Faculty of Medicine and Health, The University of Sydney, Camperdown, New South Wales, Australia; ^5^Centre for One Health, Guru Angad Dev Veterinary and Animal Sciences University, Ludhiana, Punjab, India; ^6^Ifakara Health Institute, Environmental Health, and Ecological Sciences Department, Morogoro, Tanzania; ^7^Division of Veterinary Public Health, Indian Veterinary Research Institute, Bareilly, Uttar Pradesh, India; ^8^Medical Entomology, NSW Health Pathology, Westmead, New South Wales, Australia

## Abstract

Japanese encephalitis virus (JEV) causes ~100,000 clinical cases and 25,000 deaths annually worldwide, mainly in Southeast Asia and the Western Pacific and mostly in children. JEV is transmitted to humans through the bite of mosquitoes that have fed on competent hosts. Abiotic factors, such as seasonal rainfall, influence transmission. Transmission models have an important role in understanding disease dynamics and developing prevention and control strategies to limit the impact of infectious diseases. Our goal was to investigate how transmission models capture JEV infection dynamics and their role in predicting and controlling infection. This was achieved by identifying published JEV transmission models, describing their features and identifying their limitations, to guide future modelling. A Preferred Reporting Items for Systematic Reviews and Meta-Analyses Extension for Scoping Reviews (PRISMA-ScR)-guided scoping review of peer-reviewed JEV transmission models was conducted. Databases searched included PubMed, ProQuest, Scopus, Web of Science and Google Scholar. Of the 881 full-text papers available in English, 29 were eligible for data extraction. Publication year ranged from 1975 to 2023. The median number of host populations represented in each model was 3 (range: 1–8; usually humans, mosquitoes and pigs). Most (72% [*n* = 21]) models were deterministic, using ordinary differential equations to describe transmission. Ten models were applied (representing a real JEV transmission setting) and validated with field data, while the remaining 19 models were theoretical. In the applied models, data from only a small proportion of countries in Southeast Asia and the Western Pacific were used. Limitations included gaps in knowledge of local JEV epidemiology, vector attributes and the impact of prevention and control strategies, along with a lack of model validation with field data. The lack and limitations of models highlight that further research to understand JEV epidemiology is needed and that there is opportunity to develop and implement applied models to improve control strategies for at-risk populations of animals and humans.

## 1. Introduction

Japanese encephalitis (JE) is the leading form of human acute viral encephalitis in Asia and the Pacific, and it is estimated that more than 1.5 billion people live in areas suitable for endemic JE [1, 2]. JE is caused by Japanese encephalitis virus (JEV), a zoonotic mosquito-borne orthoflavivirus. In reported human cases, the estimated case fatality approaches 30%, and of those who survive, an estimated 46% suffer permanent neurological sequelae [3]. Studies of the global burden of JE estimated 100,308 cases (95% CI: 61,720–157,522) and 25,125 deaths (95% CI: 14,550–46,031) in 2015 with most cases occurring in children aged 0–14 years (incidence: 5.4 per 100,000) [4, 5]. However, it is likely that reported case numbers are inaccurate due to inadequate data collection and diagnosis or attributed incorrectly due to cross-reactivity of serological tests with other flaviviruses [6].

It is generally accepted that JEV is transmitted from a wild reservoir host (such as ardeid birds, i.e. herons and bitterns) or an amplifying host (such as wild or domestic pigs) to humans through the bite of mainly *Culex* spp. mosquitoes [7–9]. An experimental study showed that vector-free transmission between pigs can occur via oronasal infection; however, this is yet to be reported under field conditions [10]. The epidemiology of JEV might differ between regions based on variation in the infection ecology, particularly the diversity, abundance and composition of animal (nonhuman mammals and birds) and vector communities and the circulating JEV genotype. Chicks and ducklings develop viremia [11], and JEV outbreaks have been strongly associated with chicken density [12], but further investigations are required to determine if poultry are competent hosts. Cattle, horses and dogs, like humans, have insufficient viremia to infect susceptible mosquitoes and are noncompetent hosts for JEV [13, 14]. Mosquito host feeding preferences and changes in the ratio of competent hosts to noncompetent hosts—for example, in communities with livestock—can impact JEV transmission [15]. Two epidemiological patterns of JEV have been described: endemic activity in tropical regions and epidemic activity in temperate and subtropical regions [7].

Disease transmission models are data-driven mathematical approaches to understand the parameters responsible for the dynamics of pathogen transmission and to assess the strategic responses to disease risk [16]. They can incorporate environmental, host and vector data to determine factors that influence the size and duration of outbreaks for vector-borne diseases such as dengue [17] and malaria [18]. These models have been valuable in the planning and evaluation of interventions, determining optimal prevention and control strategies and predicting the expected course of disease events [19]. However, both model development and assessment can vary widely and, therefore, so can model accuracy and reliability. This can be due to epistemic uncertainty (imprecise knowledge of parameters), aleatoric uncertainty (due to randomness) [20] or existing beliefs that influence model assumptions and interpretation of results [19], all of which can hinder the appropriate generation and use of model outputs, especially for decision-making.

In this scoping review, we aimed to examine how disease transmission models capture the dynamics of JEV infection and their use in prediction, prevention and control of JEV spread. To achieve this, we collated and described peer-reviewed studies that developed or applied models of JEV transmission in human, vector and animal populations. Models of JEV transmission were defined as those that made explicit hypotheses about the biological mechanisms that drive JEV infection dynamics in host and/or vector populations. We aimed to provide a baseline of current knowledge and knowledge gaps regarding JEV model development and parameterisation, host and vector population structures and virus transmission between hosts. The findings of this review provide a foundation for the development of improved models of JEV transmission to support JE prevention and control.

## 2. Method

### 2.1. Protocol

This scoping review was conducted according to the Preferred Reporting Items for Systematic Reviews and Meta-Analyses Extension for Scoping Reviews (PRISMA-ScR) guidelines [21]. The objective of this scoping review is to characterise and compare existing JEV transmission models applied to human, vector and animal populations, evaluating how effectively these models capture the dynamics of JEV infection and their use in predicting, preventing and controlling transmission under varying environmental and ecological conditions.

This scoping review was registered with the Open Science Framework (OSF) on 01 March 2024. The registration can be accessed at https://doi.org/10.17605/OSF.IO/G7R98.

The review protocol comprised three levels: Level 1, screening on title and abstract; Level 2, screening on full record; and Level 3, data extraction. The web-based review platform Sysrev [22] was used for Levels 1 and 2, and a spreadsheet in Google Sheets [23] was used for Level 3.

We use the term ‘record' to describe any bibliography citation captured in the searches. We use the term ‘model' to describe a disease transmission model that was either developed or implemented to describe or quantify the transmission of JEV in populations.

A total of 11 reviewers participated in the scoping review. Reviewers were selected based on their knowledge of JEV or disease transmission modelling and/or their experience in performing scoping reviews.

### 2.2. Eligibility

Records were eligible for inclusion if they were peer-reviewed literature, including peer-reviewed conference proceedings, for which the full text was available, published in English, in any year and from any country and contained primary research of interest in which a model of JEV transmission was developed or implemented. Theses and dissertations were excluded from eligibility because they may not undergo the same rigorous peer-reviewed process and often remain unpublished or have limited circulation. This exclusion ensures a focus on literature that meets a standardised level of peer review and publication. Models could range from representation of transmission in one host population to models that explicitly represented the spatio-temporal variability and heterogeneous contact structures in multiple populations.

Theses, dissertations and preprints were excluded. Records which described JEV statistical models (e.g. inferential models that aimed to identify and predict spatio-temporal occurrence based on risk factors or time-series models) were excluded.

### 2.3. Information Sources and Search Strategy

The literature search was conducted in January 2023, using the following combination of search terms:

i. ‘Japanese encephalitis' OR JEV
ii. AND: model
iii. AND: spread OR transmission
iv. time frame: all
v. language: ‘English'

Four electronic databases were searched: PubMed, ProQuest, Scopus and Web of Science (all databases) to provide a comprehensive search across various disciplines. A literature search, using the same criteria, was conducted via the Google Scholar search engine, in which the first 100 results were screened. The first 100 records from Google Scholar were included as a supplementary search step to ensure coverage of studies not indexed in primary databases. Search criteria terms and limitations can be seen in Supporting Information 1.

All records were exported into the citation manager software Endnote, and duplicates were removed. Records were then uploaded to Sysrev for Level 1 screening.

### 2.4. Selection of Relevant Records: Levels 1 and 2

During Level 1 (screening on title and abstract), two reviewers assessed each record. To maximise the sensitivity of identification of relevant records, records progressed to Level 2 if either reviewer assessed that the record might be eligible.

An agreement test was conducted prior to screening at Level 2 (screening on the full record), in which five reviewers screened the same randomly selected 20 records. Conflicting opinion about inclusion or exclusion of records was discussed to achieve agreement between reviewers and to refine and improve the clarity of the questions at each level. Table S1 lists the specific questions that guided the reviewers during each level of the screening process.

During Level 2, two reviewers initially assessed each record. Records were only included for charting in Level 3 if there was agreement that the record met the eligibility criteria between at least 2 reviewers. Conflicts of opinion were resolved via discussion and, if required, consultation with a third reviewer.

### 2.5. Data Items and Charting Process: Level 3

An agreement test was conducted prior to screening at Level 3, in which reviewers screened the same randomly selected five records. Conflicting opinion about inclusion or exclusion of records was discussed to achieve consensus between reviewers and to refine and improve the clarity of data extraction at Level 3. Table S1 provides the questions that reviewers utilised to clarify the data extraction process, ensuring consistency and thoroughness in capturing relevant information from each record. The questions comprised both free-form text and predetermined categories to support data extraction. There was no attempt to contact authors of the records to verify the data extracted; data extraction was performed solely from the information provided in the records and any accessible supporting information that accompanied them.

During Level 3, two reviewers initially extracted data from each record. Conflicting opinions about extracted data were discussed between each record's pair of reviewers and, if needed, a third reviewer to determine an agreement prior to synthesis of the extracted data.

Data items that were extracted included the year of publication, the type of modelling method and objective of the model (e.g. to estimate the likely impact of available interventions) and if the model was applied (reflected a real JEV transmission setting) or theoretical. If the model had been applied using field data, the location of the data origin was also included. Regions were defined using the World Health Organization regions. All information incorporated into each model was also recorded and comprised vector and host species, weather variables, control and prevention strategies, validation strategies and sensitivity analyses.

Data that were extracted about vector and host species included the number and species of populations used in the model, the compartments used to describe the structure of each population (e.g. susceptible–infected–recovered [SIR]) and the parameters used to describe change between compartments of a population and infection transmission within the model.

Weather data extracted included spatial, seasonal and temporal variations of rainfall, temperature and humidity and the impact of weather variability on JEV transmission.

Control and prevention strategy data included the type of strategy used and its impact on JEV transmission and how the strategy was incorporated in the model (e.g. a parameter used to decrease total vector abundance in the event of vector control).

### 2.6. Data Synthesis

The extracted data were synthesised narratively and categorised based on key themes such as the aims of the models, model structures and factors influencing JEV transmission. This included detailed information on human, vector and reservoir populations, as well as key parameters like the basic reproduction number (*R*_0_). Models were further examined for weather variables, geographic location and prevention and control strategies.

Data extracted from each record were compared with one another to ensure accurate synthesis. For example, descriptions of parameters were used to define each parameter rather than relying solely on parameter names, as parameter names may vary between records while representing the same underlying concept, or the same parameter names may refer to different concepts.

Quantitative data were synthesised using descriptive statistics, such as the frequency of specific species used in models or the number of models applied to different geographic regions. Additionally, thematic synthesis was applied to identify trends or gaps in modelling approaches, highlighting similarities and variations in key parameters, such as the basic reproduction number and vector–host interactions.

Results were organised in tables and visualised using figures and plots where appropriate to show trends and insights.

### 2.7. Identification of Additional and Missing Records

The titles of references in the bibliography of two records that were retained for data analysis in Level 3 were checked to identify any records missed by the search strategy. If records were potentially relevant to the study, they were included in the review process using the same methods as records identified in the initial search.

A weekly literature search alert was created using the same four electronic databases with the same combination of search terms to monitor new studies being published after the initial literature search was conducted. If newly published records identified by the search alert were potentially relevant to the study, they were included in the review process using the same methods as records identified in the initial search. The search alert ceased in April 2024.

## 3. Results

### 3.1. Screening

**Figure 1:**
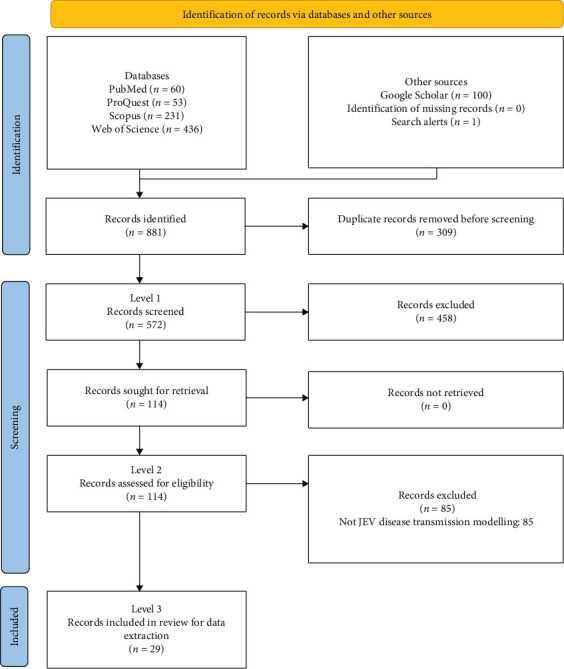
Diagram of the flow of records through the levels of a scoping review of Japanese encephalitis virus (JEV) transmission models.

Our search identified 881 records. Following removal of 309 duplicates, 572 records remained for screening (Figure 1). During Level 1 screening, 458 records were excluded, leaving 114 records for screening of full text (Level 2). Records were most commonly excluded because they were not relevant to JEV transmission modelling or were not primary literature. Overall, 29 records were included for data charting and synthesis in Level 3 (Table S1).

### 3.2. Data Charting

All 29 records included in Level 3 described single models. Six records were published in peer-reviewed conference proceedings, and the remainder were published in 22 peer-reviewed journals (Figure S1). Records were published from 1975 to 2023 (inclusive) (Figure S2).

Of the 29 models, 34% (*n* = 10) were applied to real JEV transmission settings using data such as JE incidence and pig abundance and distribution. The most frequently represented region for these models was the Western Pacific (*n* = 6) with models based in Cambodia, China, the Philippines and Taiwan. Other locations included Bangladesh, French Overseas Département of Réunion, India, Japan, Thailand and the United States (Figure S3).

### 3.3. Aims of Models

Fifteen of the models aimed to draw general conclusions about JEV transmission dynamics, such as determining equilibrium points or reproductive numbers and, in some cases, conducting stability or sensitivity analyses related to these measures [24–38]. Eight models investigated the impact of various factors influencing host and vector species on the risk of JE in humans [39–46]. Four models aimed to describe, understand and predict JE incidence [47–50]. Lastly, two models aimed to describe the effects of interventions on human, animal-reservoir or vector populations [51, 52].

### 3.4. Model Structures

Twenty-two models were deterministic. Of these, 21 were implemented using continuous time, ordinary differential equations [24–29, 31, 32, 35–37, 39–46, 51, 52], and one was implemented using discrete time, difference equations [38]. Four models were stochastic. Of these, two were implemented using continuous time, ordinary differential equations [48, 50], and two were implemented using discrete time, difference equations [47, 49]. The remaining three models were statistically converted models: two models were converted from deterministic to stochastic using geometric Brownian motion [33, 34], and one converted a deterministic model implemented with ordinary differential equations to a deterministic fractional order model [30].

Two models simulated coinfection of the human population (JEV with either *Leptospira* spp. or dengue virus) [29, 46].

One model followed a single population of feral pigs across three spatial locations, representing each individual animal within a connected network [48].

Models most commonly represented three populations—humans, vectors and an animal-reservoir (i.e. nonhuman mammal or bird)—but the number of populations in a model ranged from 1 to 8.

#### 3.4.1. Human Population

A human population was represented in 24 (83%) of the 29 models. Compartments used in models to reflect different human infection categories and the transition of the human population over time were maternal (M), vaccinated (V), susceptible (S), exposed (E), infected (I) and recovered (R) (Table S2 for description of compartments). Models described the natural history of JEV in humans using nine different model structures and the most common was SIRS (*n* = 7) (Table 1).

#### 3.4.2. Vector Population

A vector population was represented in 21 (72%) of the 29 models. Compartments used in models to reflect different vector infection categories and the transition of the vector population over time were aquatic (A), susceptible (S), exposed (E) and infected (I) (Table S2 for description of compartments). Mosquitoes were described as the main vector; however, details such as mosquito species and suitable habitat (e.g. water source and vegetation type) were not included in any models. Models described the natural history of JEV in vectors using four different model structures, and the most common was SI (*n* = 14) (Table 2).

#### 3.4.3. Animal-Reservoir Population

At least one animal-reservoir population was represented in 26 (90%) of the 29 models. Most often, only one animal-reservoir population was described in models; however, the number of populations ranged up to six. Animals were also sometimes grouped as a ‘reservoir' representing a ‘pool of infection'. The most common group listed in models was pigs (*n* = 19) followed by ‘reservoir' (*n* = 7), cattle (*n* = 2), chickens (*n* = 2), dogs (*n* = 2), ducks (*n* = 2), ‘birds' (*n* = 1) and ‘sows' (*n* = 1).

Compartments used in models to reflect different animal-reservoir infection categories and the transition of the animal-reservoir populations over time were maternal (M), vaccinated (V), susceptible (S), exposed (E), convalescent (C) and recovered (R) (Table S2 for description of compartments). Models described the natural history of JEV in animal-reservoir populations using 13 different model structures, and the most common was I (*n* = 5) followed by SIRS (*n* = 4) (Table 3).

### 3.5. State Duration Parameters and Basic Reproduction Number

**Figure 2:**
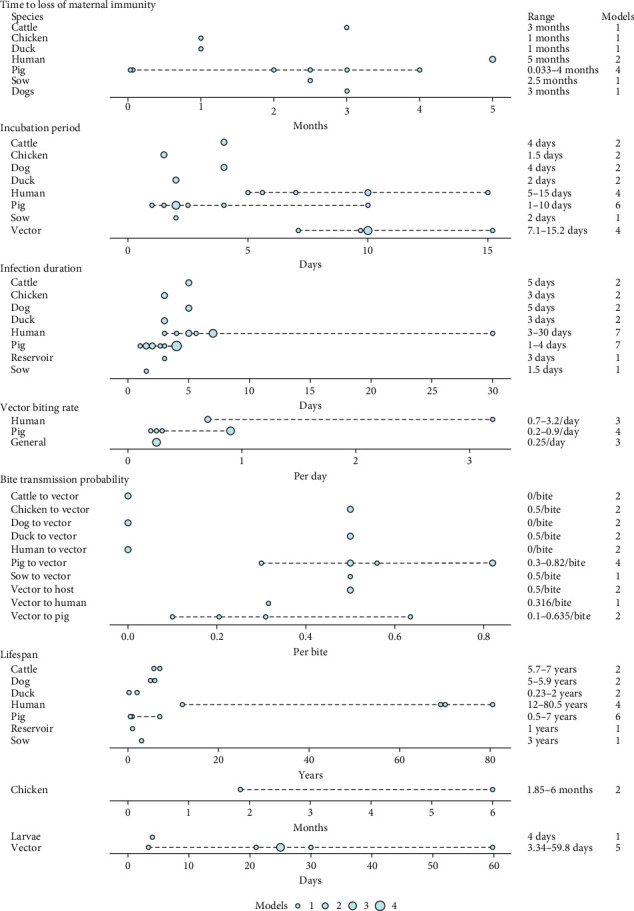
Parameter ranges (dotted lines) estimated from parameter values detailed in models when the source of data was clearly identified. The size of the point indicates the number of models with a shared parameter value.

Descriptions of parameters and parameter values were clearly presented in 21 models; however, only five models clearly stated the sources of data used to inform these parameters. In many cases, the authors of the models did not provide enough information to assess whether one or more of the parameter values used in the model were derived from literature, assumptions, unpublished field data or other sources. Units used for parameter values were not consistently identified. A total of 123 (human [*n* = 23], vector [*n* = 45] and animal-reservoir [*n* = 55]) unique parameters were identified over the 29 models. Of these parameters, 28% (*n* = 34) were accompanied with sufficient information to extract units and values (human, 39% [*n* = 9]; vector, 24% [*n* = 11]; and animal-reservoir, 25% [*n* = 14]) (Figure 2; Tables S3–S5).

**Figure 3:**
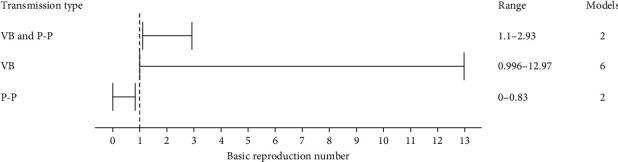
Basic reproduction number ranges estimated from six models using three transmission types. The total number of models using each transmission type is separately indicated. P-P, pig-to-pig transmission; VB, vector-borne transmission.

Basic reproduction number values were clearly described in six of the applied models. The basic reproduction number could be further broken down into three transmission types, vector-borne transmission, pig-to-pig transmission (vector-free transmission) and combined vector-borne and pig-to-pig transmission (Figure 3; Table S6). The basic reproduction number range for pig-to-pig transmission was <1. When transmission type included vector-borne transmission, the basic reproduction number was >1 and up to 12 but more commonly between 1 and 3. One model investigated the impact of cattle on JEV transmission and estimated a basic reproduction number of 1.008 in the presence of cattle and 12.97 in the absence of cattle [46].

### 3.6. Weather, Location and Other Factors

Eleven models included one or more parameters influenced by changes in weather or location, which in turn influenced their outputs. However, the information pertaining to weather and location often lacked specificity, with parameters either ambiguously defined or inadequately detailed. For instance, terms like ‘environmental discharges' were used in the models to describe environmental factors that contributed to the growth of reservoir and vector populations. Such discharges included a range of sources, such as household waste, open sewage draining, discarded tyres and poorly ventilated houses.

The vector population size was influenced in all 11 models that considered environmental factors. Five models included a ‘vector carrying capacity', which represents the maximum vector population size sustainable in a given environment [25, 32, 37, 43, 44]. Weather variations influenced parameters in five models, leading to changes in vector population size [36, 41, 48–50]. Human-induced ‘environmental discharges' influenced parameters in three models, resulting in changes to the vector population size [30, 43, 44] whilst the parameters of one model allowed for the vector population size to vary by geographic location [48].

Three models were influenced by human-induced ‘environmental discharges', similar to those impacting the vector population, which consequently led to changes in the animal-reservoir population size [30, 43, 44]. Furthermore, the parameters of one model varied based on the species and abundance of animals (birds), influenced by geographic location and time of year [48]. One model quantified animal (pig) population size by the daily consumption of pigs [50]. The authors also linked the decrease in pig abundance to a decrease in pig rearing licences.

### 3.7. Prevention and Control Strategies

Twenty of the 29 models included parameters on prevention and control strategies with 18 models listing the influence of these parameters on model output. Seven models chose to apply prevention and control strategies through direct adjustment of the parameter that the strategy was anticipated to influence. Examples included reducing mosquito population size (insecticides), reducing bite and transmission rates (mosquito nets), reducing the size of the susceptible population (vaccinating the animal-reservoir or human populations) or reducing the growth of vector and animal-reservoir populations (by decreasing human induced ‘environmental discharges') [29, 31, 35, 38, 43, 48, 49].

In contrast, some authors explicitly modelled prevention and control strategies and included a specific parameter in the model to represent the strategy. Eleven models included parameters on the vaccination rate of susceptible humans or treatment rate of JEV-infected humans [25–27, 29, 32, 34–36, 39, 40, 52], nine models included parameters on the vaccination rate of susceptible reservoir-animal populations or treatment of JEV-infected reservoir-animal populations [26, 27, 32, 36, 38, 39, 50–52], and four models included an insecticide control parameter which influenced the total mosquito population [27, 32, 36, 52]. Six models evaluated the effectiveness and cost-effectiveness of various prevention and control strategies to recommend best approaches [27, 32, 36, 39, 41, 52]. Lastly, two models assessed the impact of changing the proportions of competent and noncompetent hosts within a community [41, 46], and one explored the use of dogs as sentinel surveillance for JEV circulation [41].

### 3.8. Identified Limitations

Limitations were identified in 11 models. The authors of six records noted that their models did not include real-life variation that might influence outputs, such as the seasonality, heterogeneity and spatial distributions of populations and inclusion of various JEV transmission scenarios [28, 38, 45, 46, 46, 50]. The authors of four records noted that the numbers in the field data used to validate their model might have been under-reported (e.g. pig population data) or over-reported (e.g. the use of JEV case data) [41, 47, 48, 51]. Additionally, the authors of four models noted that there was limited information on contact structures between populations and there was limited host and vector attribute information, such as mosquito host feeding preferences, biting rates and competency as a JEV vector. The authors also noted that parameters were made to fit the geographic scale of the model and that parameters were based on collected field data when an endemic state existed. Therefore, model parameters might not be appropriate when models are scaled up to cover a larger geographic region or when annual variations in JEV transmission occur [28, 41, 45]. The epidemiology was also uncertain, such as the unknown impact that host species other than those commonly modelled can have in contributing to or limiting the spread of JEV or JEV introduction into susceptible populations [28, 45, 51].

### 3.9. Sensitivity Analyses

Sensitivity analysis was clearly described in nine models. The method used varied between models (normalised forward sensitivity index [*n* = 6], next-generation method [*n* = 2] and Morris method [*n* = 1]). Not all parameters were included in sensitivity analyses, and they were selected based on the objectives and research question. The median number of parameters assessed was 8 (range: 1–51).

All nine models' sensitivity analyses assessed the influence of input parameters on the basic reproduction number [26, 28, 29, 32–34, 40–42]. Most models assessed the impact of vector parameters on the basic reproduction number, finding that the vector biting rate, contact rate, death rate, population size and probability of infection from a competent host were the most influential. Sensitivity analyses of two of these models quantified the influence of parameters and found that those related to the force of infection for vectors and hosts—specifically the vector biting rate, vector death rate and the number of vectors—contributed the most to the total variance in basic reproduction number [28, 41].

One model assessed the influence of input parameters on the maximum number of infectious pigs [28]. It found that the the number of vectors, the recovery rate of infected pigs, the rate of loss of maternal immunity of piglets and their interactions contributed the most to the total variance in the maximum number of infectious pigs.

## 4. Discussion

Models varied widely in their structure, including combinations of vector, animal-reservoir and human populations, with differing compartmental structures selected to describe aspects of the natural history of JEV infection in those populations. While this variation sometimes aligned with specific modelling goals, such as Zahid and Kribs [46], in which the focus was on understanding the impact of cattle on joint occurrence of JE and leptospirosis, it also likely reflects great uncertainty about JEV epidemiology.

In their review on the ecology of JEV, Mulvey et al. [53] noted the detection of JEV in various domestic animals and wildlife beyond ardeid wading birds and pigs. While pigs are recognised as amplifying hosts, overlooking other potential competent hosts may lead to underestimating the true extent of JEV transmission [54, 55]. Notably, JEV transmission in animal hosts has been observed to continue despite the phasing out of pig farming in Singapore over 30 years ago [56]. Additionally, JEV has been associated with chicken density [12]. These observations suggest that other competent hosts, such as chickens, can sustain JEV circulation in regions with low pig densities, highlighting the potential importance of considering a broader range of hosts in understanding and controlling JEV transmission, especially in pig-free communities—this was reflected in few models in the current review, and nonspecifically investigated JEV circulation in species other than ardeid birds and pigs except Ladreyt et al. [41, 42].

Few of the modelling structures in the reviewed records allowed for simultaneous consideration of various factors influencing JEV spread, such as intrinsic incubation periods (e.g. in pigs) and extrinsic incubation periods (in vectors), while also enabling the assessment of different intervention strategies, like vaccination of pigs (Table 3). Despite the use of various compartmental structures and parameters, there was an absence of mosquito species-specific details in the vector populations in the models in the current review. Mosquito behaviour and ecological and host preferences vary between species known to transmit JEV [57]. Together with host competence, this variation explains the potential importance of the composition of animal populations in JEV transmission models.

Field data can inform model structure and parameter values, but the current review showed that such data were used in only 34% of the models. Integrating field data can enhance the accuracy of important parameter estimates which can improve model predictions [58]. However, field data can vary due to collection methods, such as light or CO_2_ baited traps, and surrounding environmental factors, resulting in only a limited subset of species or populations being collected, which may not fully represent broader ecological dynamics. This introduces challenges in extrapolating local data to larger regions and potentially introduces errors and assumptions into models [59, 60]. Although the integration of field data might overcome some epistemic uncertainty in model structure, epistemic uncertainty still existed with parameterisation because clarity regarding data sources and consistency in specifying units and time frames were limited. The identified discrepancies in parameter reporting led to challenges in model interpretation and comparison, highlighting the need for standardised reporting practices [19, 61]. Improving transparency and consistency in parameter reporting will be crucial for enhancing the reliability and utility of JEV transmission models.

Six of the models based on field data described vector-borne basic reproduction numbers with wide variability which reflected differences in model structure and geographic origin of data, as well as factors like population density, seasonality and vector mortality, which influence contact rates [62]. Wide variability in basic reproduction numbers has also been observed in models of other diseases; for example, measles transmission was represented with >20 different basic reproduction numbers, ranging from 5.4 to 18 [63], and caution is warranted in interpreting these values beyond their region of calculation [64]. Although the reviewed models might not accurately reflect JEV transmission dynamics overall due to substantial differences in host and vector population structures across regions, calculating basic reproduction numbers in these models can help inform risk mitigation strategies. Changes in the basic reproduction number before and after interventions can indicate their effectiveness, even if the absolute predictions are not entirely accurate. For example, a significant reduction in basic reproduction number following an intervention suggests that the intervention is likely useful.

The authors of nine of the reviewed models conducted sensitivity analyses, mainly focusing on parameters influencing basic reproduction numbers. Notably, vector dynamics-related parameters like death rate, biting rate and population size consistently emerged as significant influencers. Similar impacts have been observed in studies on diseases like African horse sickness [62] and malaria [65]. Tennant and Recker [66] highlighted the importance of obtaining field-relevant and species-dependent vector mortality rates for accurate modelling. However, most authors of reviewed models omitted sensitivity analysis, limiting readers' understanding of parameter influences and potentially compromising the utility and robustness of model outputs.

The findings from models implementing prevention and control strategies underscore the complexities inherent in planning interventions for JEV transmission. While strategies targeting human populations successfully reduced human JE cases, those aimed at animal-reservoir and vector populations, such as pig vaccination and mosquito control, interrupted the JEV transmission cycle more comprehensively. The interconnectivity of model populations underscores the necessity for integrated prevention and control approaches. However, a gap remains in employing finer parameters to evaluate specific JEV control strategies and their effectiveness. This approach has been successfully used in dengue transmission models to assess their own control strategies [17]. For instance, instead of solely introducing a parameter to reduce mosquito abundance and inferring potential strategies like using insecticide sprays, models could offer a more nuanced understanding to enhance the precision of intervention strategies. Dengue transmission models have investigated the impact of chemical, biological and environmental control methods to reduce mosquito numbers, as well as the long-term advantages and disadvantages of each. However, it is noteworthy that while dengue transmission is driven by only a few mosquito species (such as *Aedes aegypti* and *Aedes albopictus*) with highly specific habitat associations [67], JEV may have a greater number of mosquito species as potential vectors associated with a wider range of habitats [68].

The results of the 11 models that included the influence of environmental, weather and geographic factors highlight the complex and multifaceted nature of JEV transmission. According to the model outputs, vector carrying capacity, weather variations, human-induced environmental discharges and geographic location significantly impact vector and animal-reservoir abundance. Geographic and temporal variations have also been incorporated in other models to understand variations in mosquito-borne diseases [69], and human JE cases have been linked to changes in meteorological factors such as daily rainfall [70]. Models incorporating environmental drivers of JEV transmission, particularly climate features, might more accurately predict JE occurrence.

The limitations highlighted by the authors of the reviewed models reiterated the challenges in JEV transmission modelling that are identified above. Several models overlooked factors such as seasonal and spatial impacts, population heterogeneity and diverse JEV transmission scenarios. The authors also expressed concerns regarding the accuracy of field data used for model validation, citing instances of both under- and over-reporting of data, highlighting the need for reliable and more accurate data sources. Addressing these limitations is essential for refining model inputs and advancing future JEV transmission models.

The findings from this review hold significance relevant to key groups, including public health officials, veterinary professionals and local communities. By recognising the complexities of JEV transmission models, these stakeholders can improve their understanding of the disease dynamics, which is crucial for developing effective prevention and control strategies tailored to their specific contexts. This insight can inform decision-making processes and lead to more targeted intervention that address the unique epidemiological challenges faced by different communities.

This scoping review has several limitations. First, only peer-reviewed literature in English was included, which may have led to the exclusion of relevant studies published in other languages, potentially limiting geographical representation of the results. Second, the search was conducted across a limited number of databases, and whilst the first 100 records from Google Scholar were included to identify records that were not indexed in these databases, this might have led to an incomplete capture of all available literature. Finally, variations in study design and modelling methods in the included records made it challenging to directly compare results across studies. These limitations should be considered when interpreting the findings of this review.

## 5. Conclusions

Overall, this review provides insight into the literature on JEV transmission modelling, revealing both progress and challenges in understanding and mitigating the impact of JE occurrence. Despite the significant global burden of JE, it is notable that only a limited number of models have been developed to study the viral transmission dynamics. Our analysis revealed that existing models often lack comprehensive evaluation of the various transmission pathways and ecological factors influencing JEV spread. This indicates a clear gap in our understanding of current prevention and control strategies, as well as preparedness for JEV emergence in new regions. This review underscores the importance of developing robust modelling tools that can inform decision-making in JEV prevention and control, with the aim of reducing the global burden of JEV and safeguarding the health of populations at risk.

## Figures and Tables

**Table 1:** Number and types of model structures for human populations.

Model structure		Citation
SIRS	(*n* = 7)	[24, 31, 32, 35–37, 52]
SIS	(*n* = 4)	[30, 33, 43, 44]
VSIS	(*n* = 4)	[25, 34, 39, 40]
SEIR	(*n* = 3)	[42, 47, 49]
VSIRS	(*n* = 2)	[26, 27]
I	(*n* = 1)	[50]
MSEIR	(*n* = 1)	[41]
SIR	(*n* = 1)	[46]
VSIR	(*n* = 1)	[29]

Abbreviations: E, exposed; I, infected; M, maternal; R, recovered; S, susceptible; V, vaccinated.

**Table 2:** Number and types of model structures for vector populations.

Model structure		Citation
SI	(*n* = 14)	[24–27, 29, 32, 36, 37, 39, 40, 43, 45, 46, 52]
SEI	(*n* = 3)	[28, 41, 42]
ASI	(*n* = 2)	[30, 44]
I	(*n* = 2)	[33, 34]

Abbreviations: A, aquatic; E, exposed; I, infected; S, susceptible.

**Table 3:** Number and types of model structures for animal-reservoir populations.

Model structure		Animal types	Citation
I	(*n* = 5)	Birds, pigs and reservoir	[30, 32, 33, 43, 44]
SIRS	(*n* = 4)	Reservoir	[31, 35–37]
SI	(*n* = 3)	Pigs	[24, 29, 52]
SIR	(*n* = 3)	Pigs	[38, 45, 46]
SIS	(*n* = 3)	Pigs	[25, 26, 40]
MSEIR	(*n* = 1)	Cattle, chickens, dogs, ducks, pigs and sows	[41]
MSIR	(*n* = 1)	Pigs	[28]
MVSEIR	(*n* = 1)	Pigs	[51]
SEI	(*n* = 1)	Pigs	[48]
SEICR	(*n* = 1)	Pigs	[50]
SEIR	(*n* = 1)	Cattle, chickens, dogs, ducks and pigs	[42]
VSI	(*n* = 1)	Pigs	[27]
VSIS	(*n* = 1)	Pigs	[39]

Abbreviations: C, convalescent; E, exposed; I, infected; M, maternal; R, recovered; S, susceptible; V, vaccinated.

## Data Availability

All data supporting the findings of this review are included in the main article and the supporting information.

## Supporting Information

Supporting Information Figure S1: Distribution of records published in conference proceedings and journals. Figure S2: Distribution of records published by year of publication. Figure S3: Countries where Japanese encephalitis virus has been identified and countries from which field data were obtained to be used in Japanese encephalitis virus disease transmission models. Table S1: Forms used at each level of the scoping review. Table S2: Disease compartments and use within model structures. Tables S3–S6: Model parameters and value ranges. Table S6: Identified basic reproduction numbers.

## References

[1] Erlanger T. E., Weiss S., Keiser J., Utzinger J. C., Wiedenmayer K. (2009). Past, Present, and Future of Japanese Encephalitis. *Emerging Infectious Diseases*.

[2] Moore S. M. (2021). The Current Burden of Japanese Encephalitis and the Estimated Impacts of Vaccination: Combining Estimates of the Spatial Distribution and Transmission Intensity of a Zoonotic Pathogen. *PLoS Neglected Tropical Diseases*.

[3] Cheng Y., Tran Minh N., Tran Minh Q., Khandelwal S., Clapham H. E., Viennet E. (2022). Estimates of Japanese Encephalitis Mortality and Morbidity: A Systematic Review and Modeling Analysis. *PLoS Neglected Tropical Diseases*.

[4] Quan T. M., Tran T. N. T., Nguyen M. D., Tran M. N., Hannah C. (2020). Estimates of the Global Burden of Japanese Encephalitis and the Impact of Vaccination From 2000–2015. *eLife*.

[5] Campbell G., Hills S., Fischer M. (2011). Estimated Global Incidence of Japanese Encephalitis. *Bulletin of the World Health Organization*.

[6] Maeki T., Tajima S., Ikeda M. (2019). Analysis of Cross-Reactivity Between Flaviviruses With Sera of Patients With Japanese Encephalitis Showed the Importance of Neutralization Tests for the Diagnosis of Japanese Encephalitis. *Journal of Infection and Chemotherapy*.

[7] van den Hurk A. F., Ritchie S. A., Mackenzie J. S. (2009). Ecology and Geographical Expansion of Japanese Encephalitis Virus. *Annual Review of Entomology*.

[8] de Wispelaere M., Desprès P., Choumet V. (2017). European Aedes Albopictus and Culex Pipiens are Competent Vectors for Japanese Encephalitis Virus. *PLoS Neglected Tropical Diseases*.

[9] Faizah A. N., Kobayashi D., Amoa-Bosompem M. (2020). Evaluating the Competence of the Primary Vector, *Culex Tritaeniorhynchus*, and the Invasive Mosquito Species, *Aedes Japonicus Japonicus*, in Transmitting Three Japanese Encephalitis Virus Genotypes. *PLoS Neglected Tropical Diseases*.

[10] Ricklin M. E., García-Nicolás O., Brechbühl D. (2016). Vector-Free Transmission and Persistence of Japanese Encephalitis Virus in Pigs. *Nature Communications*.

[11] Cleton N. B., Bosco-Lauth A., Page M. J., Bowen R. A. (2014). Age-Related Susceptibility to Japanese Encephalitis Virus in Domestic Ducklings and Chicks. *The American Society of Tropical Medicine and Hygiene*.

[12] Walsh M. G., Pattanaik A., Vyas N. (2022). High-Risk Landscapes of Japanese Encephalitis Virus Outbreaks in India Converge on Wetlands, Rain-Fed Agriculture, Wild Ardeidae, and Domestic Pigs and Chickens. *International Journal of Epidemiology*.

[13] Boyer S., Benoit D., Sony Y. (2021). Host-Feeding Preference and Diel Activity of Mosquito Vectors of the Japanese Encephalitis Virus in Rural Cambodia. *Pathogens*.

[14] Yang D.-K., Kim B.-H., Kweon C.-H. (2008). Serosurveillance for Japanese Encephalitis, Akabane, and Aino Viruses for Thoroughbred Horses in Korea. *Journal of Veterinary Science*.

[15] Marini G., Rosá R., Pugliese A., Heesterbeek H. (2017). Exploring Vector-Borne Infection Ecology in Multi-Host Communities: A Case Study of West Nile Virus. *Journal of Theoretical Biology*.

[16] Becker A. D., Grantz K. H., Hegde S. T., Bérubé S., Cummings D. A. T., Wesolowski A. (2021). Development and Dissemination of Infectious Disease Dynamic Transmission Models During the COVID-19 Pandemic: What Can We Learn From Other Pathogens and How Can We Move Forward?. *The Lancet Digital Health*.

[17] Ogunlade S. T., Meehan M. T., Adekunle A. I., McBryde E. S. (2023). A Systematic Review of Mathematical Models of Dengue Transmission and Vector Control: 2010–2020. *Viruses*.

[18] Mandal S., Sarkar R. R., Sinha S. (2011). Mathematical Models of Malaria—a Review. *Malaria Journal*.

[19] Garnett G. P., Cousens S., Hallett T. B., Steketee R., Walker N. (2011). Mathematical Models in the Evaluation of Health Programmes. *The Lancet*.

[20] Penn M. J., Laydon D. J., Penn J. (2023). Intrinsic Randomness in Epidemic Modelling Beyond Statistical Uncertainty. *Communications Physics*.

[21] Tricco A. C., Lillie E., Zarin W. (2018). PRISMA Extension for Scoping Reviews (PRISMA-ScR): Checklist and Explanation. *Annals of Internal Medicine*.

[22] Bozada T., Borden J., Workman J., Del Cid M., Malinowski J., Luechtefeld T. (2021). Sysrev: A FAIR Platform for Data Curation and Systematic Evidence Review. *Frontiers in Artificial Intelligence*.

[23] Google (2024). Google Sheets: Online Spreadsheet Editor | Google Workspace. https://workspace.google.com/products/sheets/.

[24] Baniya V., Keval R. (2021). A Comparative Series Solutions of Japanese Encephalitis Model Using Differential Transform Method and Variational Iteration Method. *Heat Transfer*.

[25] Baniya V., Keval R. (2021). The Impact of Time Delay on the Transmission of Japanese Encephalitis Model Without Vaccination. *Proyecciones (Antofagasta)*.

[26] Baniya V., Keval R. (2020). The Influence of Vaccination on the Control of JE With a Standard Incidence Rate of Mosquitoes, Pigs and Humans. *Journal of Applied Mathematics and Computing*.

[27] De A., Maity K., Jana S., Maiti M. (2016). Application of Various Control Strategies to Japanese Encephalitic: A Mathematical Study With Human, Pig and Mosquito. *Mathematical Biosciences*.

[28] Diallo A. O. I. I., Chevalier V., Cappelle J., Duong V., Fontenille D., Duboz R. B. (2018). How Much Does Direct Transmission Between Pigs Contribute to Japanese Encephalitis Virus Circulation? A Modelling Approach in Cambodia. *PLoS ONE*.

[29] Dwivedi A., Keval R., Baniya V. (2022). A Mathematical Study of Dynamical Model for Japanese Encephalitis-Dengue Co-Infection Using JE Vaccine. *International Journal of Mathematical Modelling and Numerical Optimisation*.

[30] Ghassabzade F. A., Bagherpoorfard M. Mathematical Analysis of a Novel Japanese Encephalitis Fractional Model.

[31] Ghosh A. K., Tapaswi P. K. (1999). Dynamics of Japanese Encephalitis—A Study in Mathematical Epidemiology. *Mathematical Medicine and Biology*.

[32] Goswami N. (2022). Sensitivity and Optimal Control Analysis of Japanese Encephalitis Disease: A Mathematical Model. *Advances in Systems Science and Applications*.

[33] Kalita B., Devi A. (2020). Japanese Encephalitis From Two Outsources: A Mathematical Modeling. *Journal of Critical Reviews*.

[34] Kalita B., Devi A. (2020). Mathematical Modelling of Impact of Vaccination in Controlling Japanese Encephalitis. *International Journal on Emerging Technologies*.

[35] Mukhopadhyay B. B., Tapaswi P. K. (1994). An SIRS Epidemic Model of Japanese Encephalitis. *International Journal of Mathematics and Mathematical Sciences*.

[36] Panja P., Mondal S. K., Chattopadhyay J. (2018). Stability and Bifurcation Analysis of Japanese Encephalitis Model With/Without Effects of Some Control Parameters. *Computational and Applied Mathematics*.

[37] Tapaswi P. K., Ghosh A. K., Mukhopadhyay B. B. (1995). Transmission of Japanese Encephalitis in a 3-Population Model. *Ecological Modelling*.

[38] Wada Y. (1975). Theoretical Considerations on the Epidemic of Japanese Encephalitis. *Tropical Medicine*.

[39] Baniya V., Keval R. (2020). Mathematical Modeling and Stability Analysis of Japanese Encephalitis. *Advanced Science, Engineering and Medicine*.

[40] Baniya V., Keval R., Mohapatra R. N., Yugesh S., Kalpana G., Kalaivani C. (2021). Sensitivity and Stability Analysis in the Transmission of Japanese Encephalitis With Logistic Growing Mosquito Population. *Mathematical Analysis and Computing*.

[41] Ladreyt H., Chevalier V., Durand B., Viennet E. (2022). Modelling Japanese Encephalitis Virus Transmission Dynamics and Human Exposure in a Cambodian Rural Multi-Host System. *PLoS Neglected Tropical Diseases*.

[42] Ladreyt H., Garros C., Habchi-Hanriot N. (2023). Modelling the Potential Human Exposure to Japanese Encephalitis Virus (JEV) in Case of Introduction into Reunion Island. *Transboundary and Emerging Diseases*.

[43] Naresh R., Pandey S. (2009). Modelling and Analysis of the Spread of Japanese Encephalitis With Environmental Effects. *Applications and Applied Mathematics: An International Journal*.

[44] Ndaïrou F., Area I., Torres D. F. M. (2020). Mathematical Modeling of Japanese Encephalitis Under Aquatic Environmental Effects. *Mathematics*.

[45] Sota T., Mogi M. Models for JE Transmission Dynamics With Vector Mosquito Dynamics.

[46] Zahid M. H., Kribs C. M. (2021). Impact of Cattle on Joint Dynamics and Disease Burden of Japanese Encephalitis and Leptospirosis. *Mathematical Biosciences and Engineering*.

[47] Riad M. H., Scoglio C. M., McVey D. S., Cohnstaedt L. W. Estimation of Parameters and Basic Reproductive Ratio for Japanese Encephalitis Transmission in the Philippines Using a Sequential Monte Carlo Filter.

[48] Riad M. H., Scoglio C. M., McVey D. S., Cohnstaedt L. W. (2017). An Individual-Level Network Model for a Hypothetical Outbreak of Japanese Encephalitis in the USA. *Stochastic Environmental Research and Risk Assessment*.

[49] Riad M. H., Scoglio C. M., Cohnstaedt L. W., McVey D. S. Short-Term Forecast and Dual State-Parameter Estimation for Japanese Encephalitis Transmission Using Ensemble Kalman Filter.

[50] Zhao S., Lou Y., Chiu A. P. Y., He D. (2018). Modelling the Skip-and-Resurgence of Japanese Encephalitis Epidemics in Hong Kong. *Journal of Theoretical Biology*.

[51] Khan S. U., Salje H., Hannan A. (2014). Dynamics of Japanese Encephalitis Virus Transmission Among Pigs in Northwest Bangladesh and the Potential Impact of Pig Vaccination. *PLoS Neglected Tropical Diseases*.

[52] Kharismawati H., Fatmawati, Windarto (2019). Optimal Control of a Mathematical Model for Japanese Encephalitis Transmission. *Journal of Physics: Conference Series*.

[53] Mulvey P., Veasna D., Sebastien B. (2021). The Ecology and Evolution of Japanese Encephalitis Virus. *Pathogens*.

[54] Le Flohic G., Porphyre V., Barbazan P., Gonzalez J.-P., Johansson M. A. (2013). Review of Climate, Landscape, and Viral Genetics as Drivers of the Japanese Encephalitis Virus Ecology. *PLoS Neglected Tropical Diseases*.

[55] Levesque Z. A., Walsh M. G., Webb C. E., Zadoks R. N., Brookes V. J. (2024). A Scoping Review of Evidence of Naturally Occurring Japanese Encephalitis Infection in Vertebrate Animals Other Than Humans, Ardeid Birds and Pigs. *PLoS Neglected Tropical Diseases*.

[56] Yap G., Lim X. F., Chan S. (2019). Serological Evidence of Continued Japanese Encephalitis Virus Transmission in Singapore Nearly Three Decades After End of Pig Farming. *Parasites & Vectors*.

[57] Zardini A., Menegale F., Gobbi A. (2024). Estimating the Potential Risk of Transmission of Arboviruses in the Americas and Europe: A Modelling Study. *The Lancet Planetary Health*.

[58] Grassly N. C., Fraser C. (2008). Mathematical Models of Infectious Disease Transmission. *Nature Reviews Microbiology*.

[59] Mwanga E. P., Ngowo H. S., Mapua S. A. (2019). F.O. Evaluation of an Ultraviolet LED Trap for Atching *Anopheles* and *Culex* Mosquitoes in South-Eastern Tanzania. Parasites. *Parasites & Vectors*.

[60] Poulin B., Lefebvre G., Muranyi-Kovacs C., Hilaire S. (2017). Mosquito Traps: An Innovative, Environmentally Friendly Technique to Control Mosquitoes. *International Journal of Environmental Research and Public Health*.

[61] Milwid R., Steriu A., Arino J. (2016). Toward Standardizing a Lexicon of Infectious Disease Modeling Terms. *Frontiers in Public Health*.

[62] Lord C. C., Woolhouse M. E. J., Heesterbeek J. A. P., Mellor P. S. (1996). Vector-Borne Diseases and the Basic Reproduction Number: A Case Study of African Horse Sickness. *Medical and Veterinary Entomology*.

[63] Guerra F. M., Bolotin S., Lim G. (2017). The Basic Reproduction Number (R_0_) of Measles: A Systematic Review. *The Lancet Infectious Diseases*.

[64] Delamater P. L., Street E. J., Leslie T. F., Yang Y. T., Jacobsen K. H. (2019). Complexity of the Basic Reproduction Number (R_0_). *Emerging Infectious Diseases*.

[65] Smith D. L., McKenzie F. E., Snow R. W., Hay S. I., Grenfell B. T. (2007). Revisiting the Basic Reproductive Number for Malaria and Its Implications for Malaria Control. *PLoS Biology*.

[66] Tennant W., Recker M. (2018). Robustness of the Reproductive Number Estimates in Vector-Borne Disease Systems. *PLoS Neglected Tropical Diseases*.

[67] Lambrechts L., Scott T. W., Gubler D. J., Halstead S. B. (2010). Consequences of the Expanding Global Distribution of *Aedes Albopictus* for Dengue Virus Transmission. *PLoS Neglected Tropical Diseases*.

[68] Van den Eynde C., Sohier C., Matthijs S., De Regge N. (2022). Japanese Encephalitis Virus Interaction With Mosquitoes: A Review of Vector Competence, Vector Capacity and Mosquito Immunity. *Pathogens*.

[69] Caldwell J. M., LaBeaud A. D., Lambin E. F. (2021). Climate Predicts Geographic and Temporal Variation in Mosquito-Borne Disease Dynamics on Two Continents. *Nature Communications*.

[70] Liu Z., Zhang Y., Tong M. X. (2020). Nonlinear and Threshold Effect of Meteorological Factors on Japanese Encephalitis Transmission in Southwestern China. *The American Journal of Tropical Medicine and Hygiene*.

[71] Laidlow T. A., Johnston E. S., Zadoks R. N. (2024). Scoping Review of Japanese Encephalitis Virus Transmission Models. *medRxiv*.

