# Supplementary material for: Scoping Review of Japanese Encephalitis Virus Transmission Models
Source: Transbound Emerg Dis. 2025 Jan 27;2025:9880670. doi: 10.1155/tbed/9880670 (PMC12016872; doi:10.1155/tbed/9880670)
Supplement: Supporting Information — Figure S1: Distribution of records published in conference proceedings and journals. Figure S2: Distribution of records published by year of publication. Figure S3: Countries where Japanese encephalitis virus has been identified and countries from which field data were obtained to be used in Japanese encephalitis virus disease transmission models. Table S1: Forms used at each level of the scoping review. Table S2: Disease compartments and use within model structures. Tables S3–S6: Model parameters and value ranges. Table S6: Identified basic reproduction numbers. [file 9880670.f1.docx]

# Supplementary Material 1

***Scoping review of Japanese encephalitis virus transmission models***

Troy A. Laidlow ^a b^, Erin S. Johnston ^c^, Ruth N. Zadoks ^a b^, Michael Walsh ^b d^, Mafalda Viana ^c^, Kerrie E. Wiley ^b d^, Balbir B. Singh ^e^, Francesco Baldini ^c f^, Himani Dhanze ^g^, Cameron Webb ^b d h^, Victoria J. Brookes ^a b^

^a^ Sydney School of Veterinary Science, Faculty of Science, The University of Sydney, Camperdown, NSW, Australia
^b^ Sydney Infectious Diseases Institute, Faculty of Medicine and Health, The University of Sydney, Camperdown, NSW, Australia
^c^ School of Biodiversity, One Health and Veterinary Medicine, University of Glasgow, Glasgow, United Kingdom
^d^ Sydney School of Public Health, Faculty of Medicine and Health, The University of Sydney, Camperdown, NSW, Australia
^e^ Centre for One Health, Guru Angad Dev Veterinary and Animal Sciences University, Ludhiana, Punjab, India
^f^ Ifakara Health Institute, Environmental Health, and Ecological Sciences Department, Morogoro, United Republic of Tanzania
^g^ Division of Veterinary Public Health, Indian Veterinary Research Institute, Izatnagar, Bareilly, India
^h^ Medical Entomology, NSW Health Pathology, Westmead, NSW, Australia


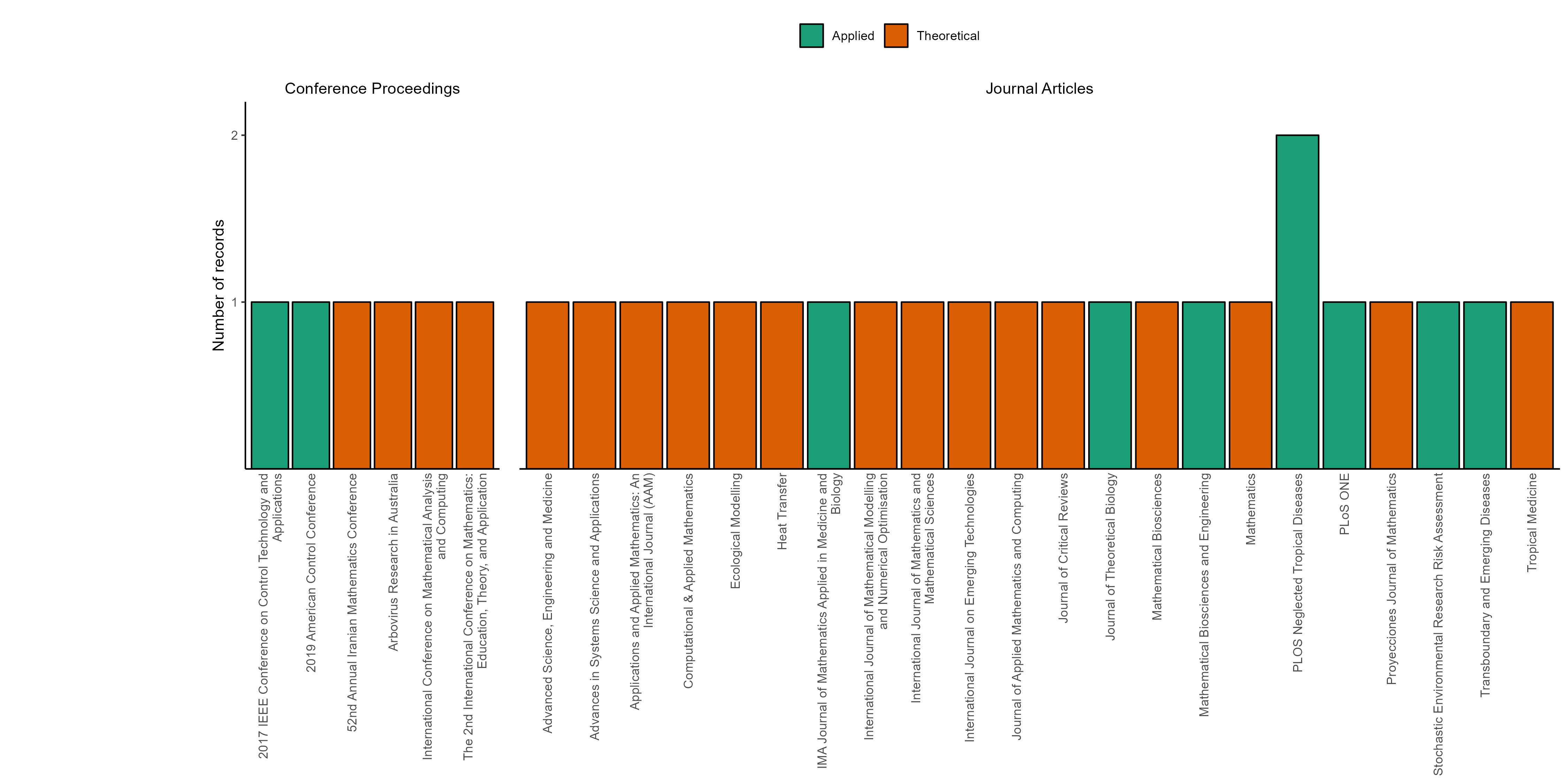


Figure S1: Distribution of peer-reviewed records published from 1975–2023 (inclusive) in conference proceedings (n=6) and journals (n=22), categorised by source, stratified by application of model to real Japanese encephalitis virus transmission settings (n=10 applied, n=19 theoretical).


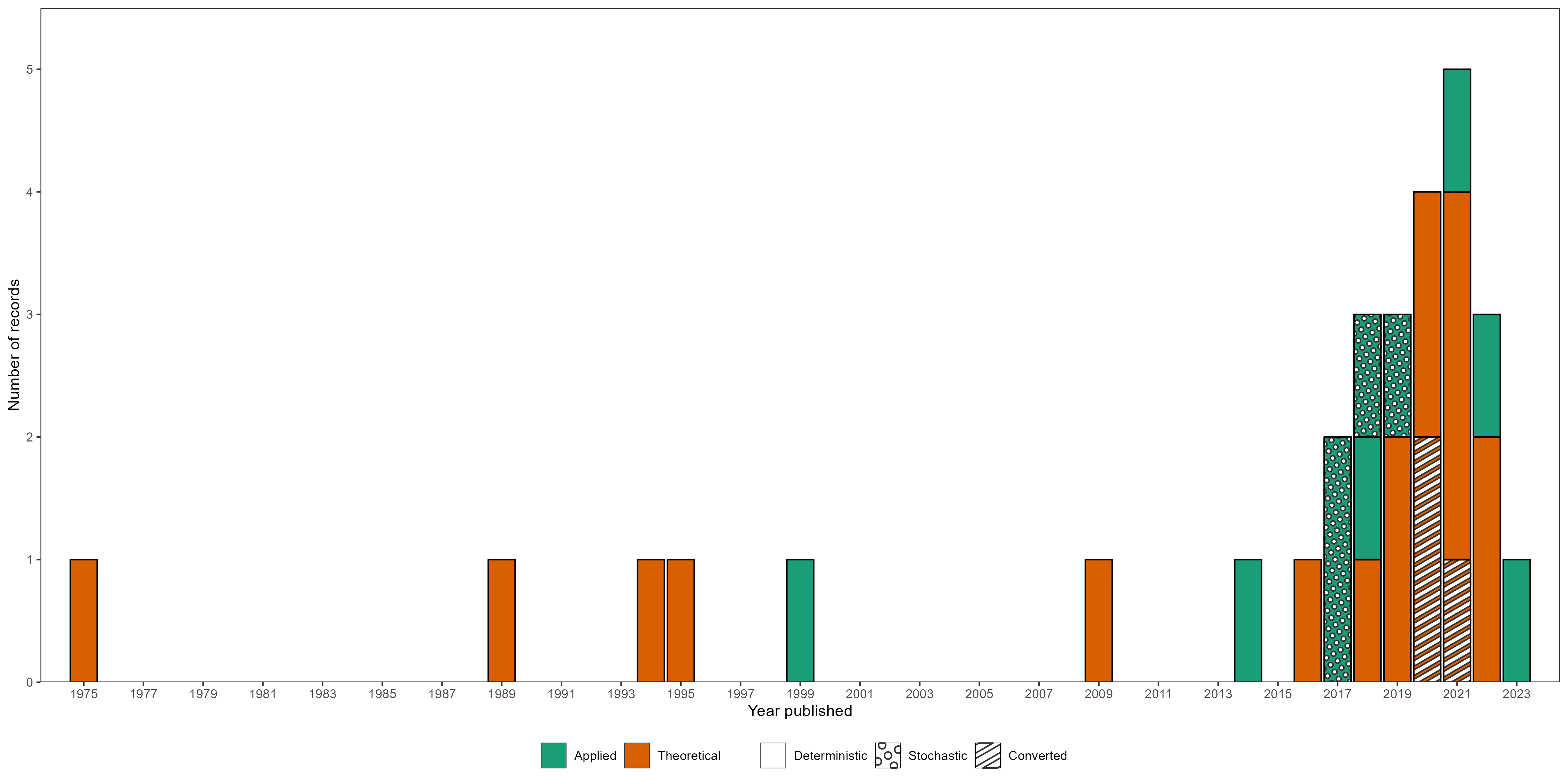


Figure S2: Distribution of records published from 1975–2023 (inclusive) by year of publication, application of model to real Japanese encephalitis virus transmission setting (n=10 applied, n=19 theoretical), and model classification as deterministic (n=22), stochastic (n=4) or converted (n=3).


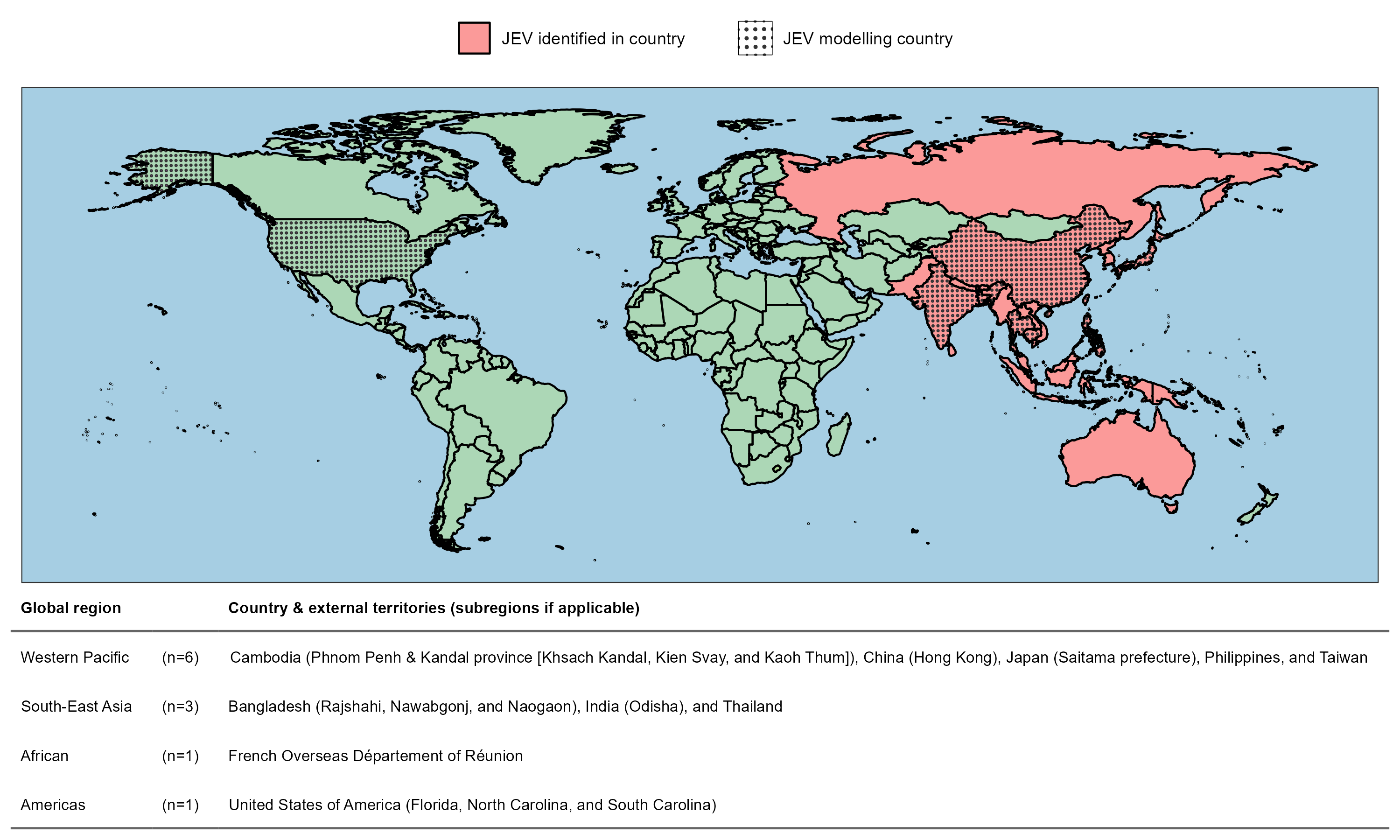


Figure S3: Countries where Japanese encephalitis virus has been identified [72] and countries from which field data were obtained to be used in Japanese encephalitis virus disease transmission models. The table below complements the map by detailing World Health Organization regions, the number of models per region, and country and external territories (including subregions where applicable) where data informed the models, noting instances where data collection and model development occurred in the same country.

Information S1: Search criteria used for databases.

**Web of Science**

| Search in: | All Databases |
| --- | --- |

|  | Topic | “Japanese encephalitis” OR JEV |
| --- | --- | --- |
| AND | Topic | model |
| AND | Topic | spread OR transmission |

| Refined By: | Languages: English |
| --- | --- |

**Scopus**

|  | Article title, Abstract, Keywords | “Japanese encephalitis” OR JEV |
| --- | --- | --- |
| AND | Article title, Abstract, Keywords | model |
| AND | Article title, Abstract, Keywords | spread OR transmission |

| Limit to: | Language: English |
| --- | --- |

**PubMed**

|  | Title/Abstract | “Japanese encephalitis” |
| --- | --- | --- |
| OR | Title/Abstract | JEV |
| AND | Title/Abstract | model |
| AND | Title/Abstract | spread |
| OR | Title/Abstract | transmission |

| Limit to: | Language: English |
| --- | --- |

**Proquest**

|  | Anywhere except full text | “Japanese encephalitis” |
| --- | --- | --- |
| AND | Anywhere except full text | model |
| AND | Anywhere except full text | spread OR transmission |

| Limit to: | Peer reviewed |
| --- | --- |
| Document type: | Article |
| Language: | English |

**Google Scholar**

Search terms: (“Japanese encephalitis” OR JEV) AND (model) AND (spread OR transmission)

| Limit to: | First 100 results |
| --- | --- |

Table S1: Forms used at each level of the scoping review

*Level 1: Screening on title and abstract*

| **Question** | **Inclusion** |
| --- | --- |
| Could this record be about the design, development, and/or implementation of a population-based JEV transmission model?  --- Choice: Yes, No | Yes |
| Are the title and abstract in English?  --- Choice: Yes, No | Yes |
| Could this record be peer-reviewed PRIMARY literature?  --- Choice: Yes, No | Yes |
| Include this article?  --- Mandatory ‘Yes’ when all other questions were ‘Yes’ | Yes |

*Level 2: Screening on full record*

| **Question** | **Inclusion** |
| --- | --- |
| Is this record about the design, development, and/or implementation of a population-based JEV transmission model  --- Choice: Yes, No | Yes |
| Is all of the content of this record in English?  --- Choice: Yes, No | Yes |
| Is this record peer-reviewed PRIMARY literature?  --- Choice: Yes, No | Yes |
| Include this article?  --- Mandatory ‘Yes’ when all other questions were ‘Yes’ | Yes |

*Level 3: Data charting*

| **Identification** |
| --- |
| Article reference |
| **Background** |
| Is this article part of conference proceedings?  --- Choice: Yes, No |
| What is the name of the journal the article is in? / What is the name of the conference proceedings? |
| What year was the study published? |
| Is the article theoretical or applied?  --- Choice: Theoretical, Applied |
| What are the aims/objectives of the model? |
| **Applied Models** |
| If the model is applied, what is the year range used in the model? |
| If the model is applied, what is the duration of model simulations? |
| If the model is applied, what is the geographic location used in model — global region? |
| --- Choice: African, Eastern Mediterranean, European, Americas, South-East Asia, and Western Pacific |
| If the model is applied, what is the geographic location used in model — country? |
| If the model is applied, what is the geographic location used in model — country subregion? |
| If the model is applied, what is the geographic location used in the model specific to country subregion, country, global region, or combination? |
| **Model Structure** |
| What type of model is being used? |
| --- Choice: Deterministic, Stochastic |
| What time step is being used in the model? |
| --- Choice: Difference, Differential, Discrete time, Continuous time |
| Have animals (non-human mammals and birds) been modelled?  --- Choice: Yes, No |
| What are the details of the animals (non-human mammals and birds) being modelled? |
| Have vectors been modelled?  --- Choice: Yes, No |
| What are the details of the vectors being modelled? |
| How many populations are represented in the model? |
| What are the compartments of the human population in the model? |
| What is the structure of the human population in the model? |
| What are the parameters used in the human population? |
| What are the compartments of the vector population in the model? |
| What is the structure of the vector population in the model? |
| What are the parameters used for the vector population? |
| What are the compartments of the animal (non-human mammals and birds) population in the model? |
| What is the structure of the animal (non-human mammals and birds) population in the model? |
| What are the parameters used for the animal (non-human mammals and birds) population? |
| What is the contact mix of the model? |
| What are the details of the contact mix in the model? |
| Have the model populations been stratified into groups? |
| What are the details of the stratified groups? |
| **Data Source** |
| What parameters used in the model are hypothetical? |
| What parameters used in the model are from literature? |
| What parameters used in the model are from data collected by authors (empirical data)? |
| What parameters used in the model are from fitting the model? |
| **Other Variables** |
| Have weather conditions been modelled?  --- Choice: Yes, No |
| What are the details of the weather conditions that have been modelled? |
| Was there ecological data (other than weather) used in the model?  --- Choice: Yes, No |
| What are the details of the ecological data (other than weather) that have been modelled? |
| Were there socio-economic factors used in the model?  --- Choice: Yes, No |
| What are the details of the socio-economic data that have been modelled? |
| Are there prevention/control measures in the model?  --- Choice: Yes, No |
| What are the details of the prevention/control measures that have been modelled? |
| What are the model outputs? |
| Was sensitivity analysis performed on the model?  --- Choice: Yes, No |
| What are the details and outputs of the sensitivity analysis? |
| **Limitations** |
| Are there any limitations identified by the author?  --- Choice: Yes, No |
| What are the details and limitations identified by the author? |

Table S2: Compartment, compartment abbreviations, and use within model structures for description of Japanese encephalitis virus (JEV) dynamics in populations.

| Compartment | Abbreviation | Description |
| --- | --- | --- |
| Aquatic | A | *Vector population*: Individuals in the early growing phase (egg, larvae, and pupae stages) who are not at risk of being infected with JEV. |
| Maternal | M | Individuals who have passive immunity due to maternal antibodies. |
| Vaccinated | V | Individuals who have immunity due to vaccination. |
| Susceptible | S | Individuals who are not infected but are at risk of becoming infected with JEV. |
| Exposed | E | Individuals who have been infected with JEV but are not yet infectious. |
| Infected | I | Individuals who are infected and infectious (capable of transmitting JEV to other individuals) |
| Convalescent | C | *Pig population*: Individuals who are infected and infectious (capable of transmitting JEV to other individuals) via oronasal secretions only. |
| Recovered | R | Individuals who have recovered from JEV infection and have gained immunity and are no longer susceptible to infection. |

Table S3: Model parameters and value ranges (when records provided sufficient information) used in representing Japanese encephalitis virus disease transmission in human populations.

| **Parameter** | **Value Range** | **Citation** |
| --- | --- | --- |
| bite transmission probability (vector-human) | 0.316 per | [46] |
| time to death (infected) | 26-120 days | [31, 46] |
| lifespan | 12-80.5 years | [31, 41, 42, 46] |
| effective contact rate (reservoir-human) | 0.001-21 | [31, 49] |
| incubation period | 5-15 days | [41, 42, 47, 49] |
| infection duration | 3-30 days | [26, 31, 41, 42, 47, 49, 49] |
| spill-over rate | 5-13 days | [50] |
| time to loss of maternal immunity | 5 months | [41, 42] |

Table S4: Model parameters and value ranges (when records provided sufficient information) used in representing Japanese encephalitis virus disease transmission in vector populations.

| **Parameter** | **Value Range** | **Citation** |
| --- | --- | --- |
| bite transmission probability (cattle-vector) | 0 per bite | [41, 42] |
| bite transmission probability (chicken-vector) | 0.5 per bite | [41, 42] |
| bite transmission probability (dog-vector) | 0 per bite | [41, 42] |
| bite transmission probability (duck-vector) | 0.5 per bite | [41, 42] |
| bite transmission probability (human-vector) | 0 per bite | [41, 42] |
| bite transmission probability (pig-vector) | 0.3-0.82 per bite | [28, 41, 42, 46] |
| bite transmission probability (sow-vector) | 0.5 per bite | [41] |
| bite transmission probability (vector-host) | 0.5 per bite | [41, 42] |
| biting rate | 0.25 per day | [41, 42, 46] |
| biting rate (human) | 0.7-3.2 per day | [26, 27, 52] |
| biting rate (pig) | 0.2-0.9 per day | [26, 27, 28, 52] |
| contact rate (vector-human) | 771250 bites/day | [46] |
| contact rate (vector-pig) | 142102 bites/day | [46] |
| lifespan | 3.34-59.8 days | [28, 41, 42, 44, 46] |
| lifespan (larvae) | 4 days | [44] |
| extrinsic incubation period | 7.1-15.2 days | [28, 38, 41, 42] |
| feeding preference (pig-cattle) | 1:1.7 pig:cattle | [41, 42] |
| feeding preference (pig-chicken) | 1:0.09 pig:chicken | [41, 42] |
| feeding preference (pig-dog) | 1:0.12 pig:dog | [41, 42] |
| feeding preference (pig-duck) | 1:0.43 pig:duck | [41, 42] |
| feeding preference (pig-human) | 1:0.5 pig:human | [41, 42] |
| feeding preference (pig-pig) | 1:1 pig:pig | [41, 42] |
| feeding preference (pig-sow) | 1:1 pig:sow | [41] |
| recruitment duration (eggs) | 1.67 days | [44] |
| vertical transmission probability | 0.04 | [46] |

Table S5: Model parameters and value ranges (when records provided sufficient information) used in representing Japanese encephalitis virus disease transmission in animal-reservoir populations.

| **Parameter** | **Value Range** | **Citation** |
| --- | --- | --- |
| bite transmission probability (reservoir-vector) | 0.00021 per bite | [44] |
| bite transmission probability (vector-pig) | 0.1-0.635 per bite | [28, 46] |
| convalescent duration (pig) | 1-4 days | [50] |
| lifespan (cattle) | 5.7-7 years | [41, 42] |
| lifespan (chicken) | 1.85-6 months | [41, 42] |
| lifespan (dog) | 5-5.9 years | [41, 42] |
| lifespan (duck) | 0.23-2 years | [41, 42] |
| lifespan (pig) | 0.5-7 years | [38, 41, 42, 46, 50, 51] |
| lifespan (reservoir) | 1 years | [31] |
| lifespan (sow) | 3 years | [41] |
| effective contact rate (vector-pig) | 0-0.4 | [50, 51] |
| effective contact rate (vector-reservoir) | 0.55-0.6 | [31] |
| external introduction proportion (imported pig) | 0.43-1.45 | [50] |
| external introduction proportion (other hosts) | 0.05 | [51] |
| incubation period (cattle) | 4 days | [41, 42] |
| incubation period (chicken) | 1.5 days | [41, 42] |
| incubation period (dog) | 4 days | [41, 42] |
| incubation period (duck) | 2 days | [41, 42] |
| incubation period (pig) | 1-10 days | [38, 41, 42, 48, 50, 51] |
| incubation period (sow) | 2 days | [41, 42] |
| infection duration (cattle) | 5 days | [41, 42] |
| infection duration (chicken) | 3 days | [41, 42] |
| infection duration (dog) | 5 days | [41, 42] |
| infection duration (duck) | 3 days | [41, 42] |
| infection duration (pig) | 1-4 days | [28, 38, 41, 42, 46, 50, 51] |
| infection duration (reservoir) | 3 days | [31] |
| infection duration (sow) | 1.5 days | [41] |
| time to loss of maternal immunity (cattle) | 3 months | [41] |
| time to loss of maternal immunity (chicken) | 1 months | [41] |
| time to loss of maternal immunity (dogs) | 3 months | [41] |
| time to loss of maternal immunity (duck) | 1 months | [41] |
| time to loss of maternal immunity (pig) | 0.033-4 months | [28, 38, 41, 51] |
| time to loss of maternal immunity (sow) | 2.5 months | [41] |

Table S6: Identified basic reproduction numbers and value ranges (when records provided sufficient information) used in representing Japanese encephalitis virus disease transmission.

| **Types of transmission** | **Value Range** | **Citation** |
| --- | --- | --- |
| Pig-pig | 0-0.83 | [28, 50] |
| Vector-borne & pig-pig | 1.1-2.93 | [28, 50] |
| Vector-borne | 0.996-12.97 | [28, 41, 46, 47, 50, 51] |

# References

1. T. E. Erlanger, S. Weiss, J. Keiser, J. C. Utzinger, and K. Wiedenmayer, “Past, Present, and Future of Japanese Encephalitis,” Emerging Infectious Diseases, vol. 15, no. 1, pp. 1–7, 2009.
2. S. M. Moore, “The Current Burden of Japanese Encephalitis and the Estimated Impacts of Vaccination: Combining Estimates of the Spatial Distribution and Transmission Intensity of a Zoonotic Pathogen,” PLoS Neglected Tropical Diseases, vol. 15, no. 10, Article ID e0009385, 2021.
3. Y. Cheng, N. Tran Minh, Q. Tran Minh, S. Khandelwal, H. E. Clapham, and E. Viennet, “Estimates of Japanese Encephalitis Mortality and Morbidity: A Systematic Review and Modeling Analysis,” PLoS Neglected Tropical Diseases, vol. 16, no. 5, Article ID e0010361, 2022.
4. T. M. Quan, T. N. T. Tran, M. D. Nguyen, M. N. Tran, and C. Hannah, “Estimates of the Global Burden of Japanese Encephalitis and the Impact of Vaccination From 2000–2015,” eLife, vol. 9, Article ID e51027, 2020.
5. G. Campbell, S. Hills, M. Fischer et al., “Estimated Global Incidence of Japanese Encephalitis,” Bulletin of the World Health Organization, vol. 89, no. 10, pp. 766–774, 2011.
6. T. Maeki, S. Tajima, M. Ikeda et al., “Analysis of Cross-Reactivity Between Flaviviruses With Sera of Patients With Japanese Encephalitis Showed the Importance of Neutralization Tests for the Diagnosis of Japanese Encephalitis,” Journal of Infection and Chemotherapy, vol. 25, no. 10, pp. 786–790, 2019.
7. A. F. van den Hurk, S. A. Ritchie, and J. S. Mackenzie, “Ecology and Geographical Expansion of Japanese Encephalitis Virus,” Annual Review of Entomology, vol. 54, no. 1, pp. 17–35, 2009.
8. M. de Wispelaere, P. Desprès, and V. Choumet, “European Aedes Albopictus and Culex Pipiens are Competent Vectors for Japanese Encephalitis Virus,” PLoS Neglected Tropical Diseases, vol. 11, no. 1, Article ID e0005294, 2017.
9. A. N. Faizah, D. Kobayashi, M. Amoa-Bosompem et al., “Evaluating the Competence of the Primary Vector, Culex Tritaeniorhynchus, and the Invasive Mosquito Species, Aedes Japonicus Japonicus, in Transmitting Three Japanese Encephalitis Virus Genotypes,” PLoS Neglected Tropical Diseases, vol. 17, no. 1, Article ID e0011052, 2023.
10. M. E. Ricklin, O. García-Nicolás, D. Brechbühl et al., “Vector-Free Transmission and Persistence of Japanese Encephalitis Virus in Pigs,” Nature Communications, vol. 7, no. 1, Article ID 10832, 2016.
11. N. B. Cleton, A. Bosco-Lauth, M. J. Page, and R. A. Bowen, “Age-Related Susceptibility to Japanese Encephalitis Virus in Domestic Ducklings and Chicks,” The American Society of Tropical Medicine and Hygiene, vol. 90, no. 2, pp. 242–246, 2014.
12. M. G. Walsh, A. Pattanaik, N. Vyas et al., “High-Risk Landscapes of Japanese Encephalitis Virus Outbreaks in India Converge on Wetlands, Rain-Fed Agriculture, Wild Ardeidae, and Domestic Pigs and Chickens,” International Journal of Epidemiology, vol. 51, no. 5, pp. 1408–1418, 2022.
13. S. Boyer, D. Benoit, Y. Sony et al., “Host-Feeding Preference and Diel Activity of Mosquito Vectors of the Japanese Encephalitis Virus in Rural Cambodia,” Pathogens, vol. 10, no. 3, Article ID 376, 2021.
14. D.-K. Yang, B.-H. Kim, C.-H. Kweon et al., “Serosurveillance for Japanese Encephalitis, Akabane, and Aino Viruses for Thoroughbred Horses in Korea,” Journal of Veterinary Science, vol. 9, no. 4, Article ID 381, 2008.
15. G. Marini, R. Rosá, A. Pugliese, and H. Heesterbeek, “Exploring Vector-Borne Infection Ecology in Multi-Host Communities: A Case Study of West Nile Virus,” Journal of Theoretical Biology, vol. 415, pp. 58–69, 2017.
16. A. D. Becker, K. H. Grantz, S. T. Hegde, S. Bérubé, D. A. T. Cummings, and A. Wesolowski, “Development and Dissemination of Infectious Disease Dynamic Transmission Models During the COVID-19 Pandemic: What Can We Learn From Other Pathogens and How Can We Move Forward?” The Lancet Digital Health, vol. 3, no. 1, pp. e41–e50, 2021.
17. S. T. Ogunlade, M. T. Meehan, A. I. Adekunle, and E. S. McBryde, “A Systematic Review of Mathematical Models of Dengue Transmission and Vector Control: 2010–2020,” Viruses, vol. 15, no. 1, Article ID 254, 2023.
18. S. Mandal, R. R. Sarkar, and S. Sinha, “Mathematical Models of Malaria—a Review,” Malaria Journal, vol. 10, Article ID 202, 2011.
19. G. P. Garnett, S. Cousens, T. B. Hallett, R. Steketee, and N. Walker, “Mathematical Models in the Evaluation of Health Programmes,” The Lancet, vol. 378, no. 9790, pp. 515–525, 2011.
20. M. J. Penn, D. J. Laydon, J. Penn et al., “Intrinsic Randomness in Epidemic Modelling Beyond Statistical Uncertainty,” Communications Physics, vol. 6, no. 1, Article ID 146, 2023.
21. A. C. Tricco, E. Lillie, W. Zarin et al., “PRISMA Extension for Scoping Reviews (PRISMA-ScR): Checklist and Explanation,” Annals of Internal Medicine, vol. 169, no. 7, pp. 467–473, 2018.
22. T. Bozada Jr, J. Borden, J. Workman, M. Del Cid, J. Malinowski, and T. Luechtefeld, “Sysrev: A FAIR Platform for Data Curation and Systematic Evidence Review,” Frontiers in Artificial Intelligence, vol. 4, Article ID 685298, 2021.
23. Google, “Google Sheets: Online Spreadsheet Editor | Google Workspace,” 2024, Google Sheets. 2024 <https://workspace.google.com/products/sheets/>.
24. V. Baniya and R. Keval, “A Comparative Series Solutions of Japanese Encephalitis Model Using Differential Transform Method and Variational Iteration Method,” Heat Transfer, vol. 50, no. 6, pp. 5885–5905, 2021.
25. V. Baniya and R. Keval, “The Impact of Time Delay on the Transmission of Japanese Encephalitis Model Without Vaccination,” Proyecciones (Antofagasta), vol. 40, no. 6, pp. 1367–1410, 2021.
26. V. Baniya and R. Keval, “The Influence of Vaccination on the Control of JE With a Standard Incidence Rate of Mosquitoes, Pigs and Humans,” Journal of Applied Mathematics and Computing, vol. 64, no. 1-2, pp. 519–550, 2020.
27. A. De, K. Maity, S. Jana, and M. Maiti, “Application of Various Control Strategies to Japanese Encephalitic: A Mathematical Study With Human, Pig and Mosquito,” Mathematical Biosciences, vol. 282, pp. 46–60, 2016.
28. A. O. I. I. Diallo, V. Chevalier, J. Cappelle, V. Duong, D. Fontenille, and R. B. Duboz, “How Much Does Direct Transmission Between Pigs Contribute to Japanese Encephalitis Virus Circulation? A Modelling Approach in Cambodia,” PLoS ONE, vol. 13, no. 8, Article ID e0201209, 2018.
29. A. Dwivedi, R. Keval, and V. Baniya, “A Mathematical Study of Dynamical Model for Japanese Encephalitis-Dengue Co-Infection Using JE Vaccine,” International Journal of Mathematical Modelling and Numerical Optimisation, vol. 12, no. 4, pp. 416–441, 2022.
30. F. A. Ghassabzade and M. Bagherpoorfard, “Mathematical Analysis of a Novel Japanese Encephalitis Fractional Model,” in 2021 52nd Annual Iranian Mathematics Conference, pp. 21–23, IEEE, Kerman, Iran, Islamic Republic of, 2021.
31. A. K. Ghosh and P. K. Tapaswi, “Dynamics of Japanese Encephalitis—A Study in Mathematical Epidemiology,” Mathematical Medicine and Biology, vol. 16, no. 1, pp. 1–27, 1999.
32. N. Goswami, “Sensitivity and Optimal Control Analysis of Japanese Encephalitis Disease: A Mathematical Model,” Advances in Systems Science and Applications, vol. 22, pp. 148–166, 2022.
33. B. Kalita and A. Devi, “Japanese Encephalitis From Two Outsources: A Mathematical Modeling,” Journal of Critical Reviews, vol. 7, no. 12, pp. 309–315, 2020.
34. B. Kalita and A. Devi, “Mathematical Modelling of Impact of Vaccination in Controlling Japanese Encephalitis,” International Journal on Emerging Technologies, vol. 11, no. 3, pp. 792–796, 2020.
35. B. B. Mukhopadhyay and P. K. Tapaswi, “An SIRS Epidemic Model of Japanese Encephalitis,” International Journal of Mathematics and Mathematical Sciences, vol. 17, Article ID 352614, 9 pages, 1994.
36. [11](https://aps.wiley.com/view.manuscript/tbed/9880670/1/)P. Panja, S. K. Mondal, and J. Chattopadhyay, “Stability and Bifurcation Analysis of Japanese Encephalitis Model With/Without Effects of Some Control Parameters,” Computational and Applied Mathematics, vol. 37, no. 2, pp. 1330–1351, 2018.
37. P. K. Tapaswi, A. K. Ghosh, and B. B. Mukhopadhyay, “Transmission of Japanese Encephalitis in a 3-Population Model,” Ecological Modelling, vol. 83, no. 3, pp. 295–309, 1995.
38. Y. Wada, “Theoretical Considerations on the Epidemic of Japanese Encephalitis,” Tropical Medicine, vol. 17, no. 1, pp. 171–199, 1975.
39. V. Baniya and R. Keval, “Mathematical Modeling and Stability Analysis of Japanese Encephalitis,” Advanced Science, Engineering and Medicine, vol. 12, no. 1, pp. 120–127, 2020.
40. V. Baniya and R. Keval, “Sensitivity and Stability Analysis in the Transmission of Japanese Encephalitis With Logistic Growing Mosquito Population,” in Mathematical Analysis and Computing, R. N. Mohapatra, S. Yugesh, G. Kalpana, and C. Kalaivani, Eds., pp. 55–69, Springer Singapore, Singapore, 2021.
41. H. Ladreyt, V. Chevalier, B. Durand, and E. Viennet, “Modelling Japanese Encephalitis Virus Transmission Dynamics and Human Exposure in a Cambodian Rural Multi-Host System,” PLoS Neglected Tropical Diseases, vol. 16, no. 7, Article ID e0010572, 2022.
42. H. Ladreyt, C. Garros, N. Habchi-Hanriot et al., “Modelling the Potential Human Exposure to Japanese Encephalitis Virus (JEV) in Case of Introduction into Reunion Island,” Transboundary and Emerging Diseases, vol. 2023, Article ID 3118640, 11 pages, 2023.
43. R. Naresh and S. Pandey, “Modelling and Analysis of the Spread of Japanese Encephalitis With Environmental Effects,” Applications and Applied Mathematics: An International Journal, vol. 4, no. 1, pp. 155–175, 2009.
44. F. Ndaïrou, I. Area, and D. F. M. Torres, “Mathematical Modeling of Japanese Encephalitis Under Aquatic Environmental Effects,” Mathematics, vol. 8, no. 11, Article ID 1880, 2020.
45. [12](https://aps.wiley.com/view.manuscript/tbed/9880670/1/)T. Sota and M. Mogi, “Models for JE Transmission Dynamics With Vector Mosquito Dynamics,” in Arbovirus Research in Australia Proceedings Fifth Symposium, pp. 144–148, EurekaMag, Brisbane, Australia, 1989.
46. M. H. Zahid and C. M. Kribs, “Impact of Cattle on Joint Dynamics and Disease Burden of Japanese Encephalitis and Leptospirosis,” Mathematical Biosciences and Engineering, vol. 18, no. 4, pp. 3046–3072, 2021.
47. M. H. Riad, C. M. Scoglio, D. S. McVey, and L. W. Cohnstaedt, “Estimation of Parameters and Basic Reproductive Ratio for Japanese Encephalitis Transmission in the Philippines Using a Sequential Monte Carlo Filter,” in 2017 IEEE Conference on Control Technology and Applications (CCTA), pp. 668–673, IEEE, Maui, HI, USA, 2017.
48. M. H. Riad, C. M. Scoglio, D. S. McVey, and L. W. Cohnstaedt, “An Individual-Level Network Model for a Hypothetical Outbreak of Japanese Encephalitis in the USA,” Stochastic Environmental Research and Risk Assessment, vol. 31, no. 2, pp. 353–367, 2017.
49. M. H. Riad, C. M. Scoglio, L. W. Cohnstaedt, and D. S. McVey, “Short-Term Forecast and Dual State-Parameter Estimation for Japanese Encephalitis Transmission Using Ensemble Kalman Filter,” in 2019 American Control Conference (ACC), pp. 3444–3449, IEEE, Philadelphia, PA, USA, 2019.
50. S. Zhao, Y. Lou, A. P. Y. Chiu, and D. He, “Modelling the Skip-and-Resurgence of Japanese Encephalitis Epidemics in Hong Kong,” Journal of Theoretical Biology, vol. 454, pp. 1–10, 2018.
51. S. U. Khan, H. Salje, A. Hannan et al., “Dynamics of Japanese Encephalitis Virus Transmission Among Pigs in Northwest Bangladesh and the Potential Impact of Pig Vaccination,” PLoS Neglected Tropical Diseases, vol. 8, no. 9, Article ID e3166, 2014.
52. H. Kharismawati, Fatmawati, and Windarto, “Optimal Control of a Mathematical Model for Japanese Encephalitis Transmission,” Journal of Physics: Conference Series, vol. 1306, Article ID 012034, 2019.
53. P. Mulvey, D. Veasna, B. Sebastien et al., “The Ecology and Evolution of Japanese Encephalitis Virus,” Pathogens, vol. 10, no. 12, Article ID 1534, 2021.
54. G. Le Flohic, V. Porphyre, P. Barbazan, J.-P. Gonzalez, and M. A. Johansson, “Review of Climate, Landscape, and Viral Genetics as Drivers of the Japanese Encephalitis Virus Ecology,” PLoS Neglected Tropical Diseases, vol. 7, no. 9, Article ID e2208, 2013.
55. Z. A. Levesque, M. G. Walsh, C. E. Webb, R. N. Zadoks, and V. J. Brookes, “A Scoping Review of Evidence of Naturally Occurring Japanese Encephalitis Infection in Vertebrate Animals Other Than Humans, Ardeid Birds and Pigs,” PLoS Neglected Tropical Diseases, vol. 18, no. 10, Article ID e0012510, 2024.
56. G. Yap, X. F. Lim, S. Chan et al., “Serological Evidence of Continued Japanese Encephalitis Virus Transmission in Singapore Nearly Three Decades After End of Pig Farming,” Parasites & Vectors, vol. 12, no. 1, Article ID 244, 2019.
57. A. Zardini, F. Menegale, A. Gobbi et al., “Estimating the Potential Risk of Transmission of Arboviruses in the Americas and Europe: A Modelling Study,” The Lancet Planetary Health, vol. 8, no. 1, pp. e30–e40, 2024.
58. N. C. Grassly and C. Fraser, “Mathematical Models of Infectious Disease Transmission,” Nature Reviews Microbiology, vol. 6, no. 6, pp. 477–487, 2008.
59. E. P. Mwanga, H. S. Ngowo, S. A. Mapua et al., “F.O. Evaluation of an Ultraviolet LED Trap for Atching Anopheles and Culex Mosquitoes in South-Eastern Tanzania. Parasites,” Parasites & Vectors, vol. 12, no. 1, Article ID 418, 2019.
60. B. Poulin, G. Lefebvre, C. Muranyi-Kovacs, and S. Hilaire, “Mosquito Traps: An Innovative, Environmentally Friendly Technique to Control Mosquitoes,” International Journal of Environmental Research and Public Health, vol. 14, no. 3, Article ID 313, 2017.
61. R. Milwid, A. Steriu, J. Arino et al., “Toward Standardizing a Lexicon of Infectious Disease Modeling Terms,” Frontiers in Public Health, vol. 4, Article ID 213, 2016.
62. C. C. Lord, M. E. J. Woolhouse, J. A. P. Heesterbeek, and P. S. Mellor, “Vector-Borne Diseases and the Basic Reproduction Number: A Case Study of African Horse Sickness,” Medical and Veterinary Entomology, vol. 10, no. 1, pp. 19–28, 1996.
63. F. M. Guerra, S. Bolotin, G. Lim et al., “The Basic Reproduction Number (R_0_) of Measles: A Systematic Review,” The Lancet Infectious Diseases, vol. 17, no. 12, pp. e420–e428, 2017.
64. P. L. Delamater, E. J. Street, T. F. Leslie, Y. T. Yang, and K. H. Jacobsen, “Complexity of the Basic Reproduction Number (R_0_),” Emerging Infectious Diseases, vol. 25, no. 1, pp. 1–4, 2019.
65. D. L. Smith, F. E. McKenzie, R. W. Snow, S. I. Hay, and B. T. Grenfell, “Revisiting the Basic Reproductive Number for Malaria and Its Implications for Malaria Control,” PLoS Biology, vol. 5, no. 3, Article ID e42, 2007.
66. W. Tennant and M. Recker, “Robustness of the Reproductive Number Estimates in Vector-Borne Disease Systems,” PLoS Neglected Tropical Diseases, vol. 12, no. 12, Article ID e0006999, 2018.
67. L. Lambrechts, T. W. Scott, D. J. Gubler, and S. B. Halstead, “Consequences of the Expanding Global Distribution of Aedes Albopictus for Dengue Virus Transmission,” PLoS Neglected Tropical Diseases, vol. 4, no. 5, Article ID e646, 2010.
68. C. Van den Eynde, C. Sohier, S. Matthijs, and N. De Regge, “Japanese Encephalitis Virus Interaction With Mosquitoes: A Review of Vector Competence, Vector Capacity and Mosquito Immunity,” Pathogens, vol. 11, no. 3, Article ID 317, 2022.
69. J. M. Caldwell, A. D. LaBeaud, E. F. Lambin et al., “Climate Predicts Geographic and Temporal Variation in Mosquito-Borne Disease Dynamics on Two Continents,” Nature Communications, vol. 12, no. 1, Article ID 1233, 2021.
70. Z. Liu, Y. Zhang, M. X. Tong et al., “Nonlinear and Threshold Effect of Meteorological Factors on Japanese Encephalitis Transmission in Southwestern China,” The American Journal of Tropical Medicine and Hygiene, vol. 103, no. 6, pp. 2442–2449, 2020.
71. T. A. Laidlow, E. S. Johnston, R. N. Zadoks et al., “Scoping Review of Japanese Encephalitis Virus Transmission Models,” medRxiv, 2024.
72. CDC, “Centers for Disease Control and Prevention, Geographic Distribution of Japanese Encephalitis Virus | Japanese Encephalitis | CDC,” 2023, <https://www.cdc.gov/japaneseencephalitis/maps/index.html>, April 7, 2023.
